# Developing a core outcome set for acetabular fractures: a systematic review protocol

**DOI:** 10.1186/s13643-024-02571-8

**Published:** 2024-06-05

**Authors:** Denise Schulz, Adrian Deichsel, Martin C. Jordan, Joachim Windolf, Michael J. Raschke, Anne Neubert

**Affiliations:** 1 Department of Orthopaedics and Traumatology, Medical Faculty and University Hospital Düsseldorf, Heinrich-Heine-University Düsseldorf, Düsseldorf, Germany; 2 TraumaEvidence @ German Society of Traumatology, Berlin, Germany; 3 Department of Trauma, Hand and Reconstructive Surgery, University Hospital Münster, University of Münster, Münster, Germany; 4 Department of Orthopaedic Traumatology, University Hospital Würzburg, Julius-Maximilian-University of Würzburg, Würzburg, Germany

**Keywords:** Core outcome set, Acetabular fractures, Systematic review, Outcome measures

## Abstract

**Background:**

Clinical trials investigating acetabular fractures are heterogeneous in their investigated outcomes and their corresponding measurements. Standardization may facilitate comparability and pooling of research results, which would lead to an increase in knowledge about the optimal treatment of acetabular fractures, resulting in long-term evidence-based treatment decisions and improvements in patient care. The aim of this systematic review is to identify the reported outcomes and their measurements from studies on treatments for acetabular fractures to develop a core outcome set which contains the most relevant outcome measures to be included in future studies.

**Methods:**

Studies published in English and German including patients aged 16 years and older, with a surgically treated acetabular fracture, will be included. Studies with nonsurgical treatment, pathologic fractures, polytraumatized patients, and patients younger than 16 years of age will be excluded because other outcomes may be of interest in these cases. Any prospective and retrospective study will be included. Systematic reviews will be excluded, but their included studies will be screened for eligibility. The literature will be searched on MEDLINE, CENTRAL, Web of Science, ClinicalTrials.gov, and WHO ICTRP. Risk of selective reporting of outcomes will be assessed using the *Outcome Reporting Bias in Trials* classification system. Heterogeneously defined outcomes that measure the same outcome will be grouped and subsequently categorized into outcome domains using the taxonomy of the *Core Outcome Measures in Effectiveness Trials Initiative*.

**Discussion:**

It is expected that a high number of studies will be included, and many outcomes will be identified using different definitions and measurement instruments. A limitation of this systematic review is that only previously investigated outcomes will be detected, thus disregarding potentially relevant outcomes.

**Systematic review registration:**

PROSPERO CRD42022357644

**Supplementary Information:**

The online version contains supplementary material available at 10.1186/s13643-024-02571-8.

## Background

Fractures of the acetabulum are rare with an incidence of less than 10 cases per 100,000 population. However, the incidence of acetabular fractures is rising, especially among elderly [1–3]. Typically, the fracture mechanism is either high energy (e.g., falls from greater heights, or motor vehicle accidents) in younger patients or low energy (e.g., simple falls) most commonly seen in elderly [4–6]. Acetabular fractures are associated with a high mortality and a high socioeconomic burden [7]. Oftentimes, these fractures require joint replacements that are commonly challenging [8].

Clinical trials are conducted to determine the safety and efficacy of an intervention. Studies may also examine which therapies are superior and reduce, for example, the mortality rate associated with acetabular fractures [9]. Clinical studies that investigate fractures of the acetabulum are commonly small and heterogenous, examining a variety of different outcomes. The heterogeneity of investigated outcomes, their definitions, and the wide variety of instruments for measuring a single outcome makes it difficult to synthesize study results. Furthermore, often scores used to assess the health status of patients with acetabular fractures are not formally validated [10]. Unifying the reporting of outcomes and their instruments in studies that investigate acetabular fractures could enhance the comparability among different studies and thereby assist to ease research aggregation by means of systematic reviews and meta-analysis. Improving the aggregated knowledge and research about how to best treat acetabular fractures could lead to improved evidence-based treatment decision and thus, better patient care.

In order to counteract the heterogeneity of investigated outcomes, core outcome sets (COS) are developed. A COS specifies an agreed minimum set of outcomes that should be measured and reported in all clinical trials or research studies in a specific area of healthcare. Furthermore, it states how each individual outcome should be measured, to improve comparability and summary of study results [9, 11]. In addition, a COS can lead to a reduction of unnecessary research and thus to economic savings through studies that measure the same outcomes [12]. For example, a COS defining key outcomes for hip fractures does already exist [13]. However, to the best of our knowledge, no COS for acetabular fractures is currently available.

### Objective

The aim of this study is to identify the outcomes, their definitions, and outcome measures used in clinical studies investigating surgical treatments of acetabular fractures. As recommended by the *Core Outcome Measures in Effectiveness Trials* (COMET) *Initiative*, this systematic review identifies existing knowledge as a first step toward developing a COS [9]. In a second step, the results of the systematic review for the development of a COS will be used in a subsequent study to inform a consensus group on potential outcomes for the COS using the Delphi method.

The research question of this systematic review is as follows: Which outcomes are reported in clinical trials investigating surgical treatment of acetabular fractures in skeletally mature patients?

#### Scope

As recommended by the *Core Outcome Set-STAndards for Development* (COS-STAD), the scope of this systematic review and the final COS was specified with the following four components [14]:

#### Research or practice setting

The results of this systematic review will be used in a subsequent study to develop the COS for application in research. The final COS will be used in studies investigating operative treatments of acetabular fractures.

#### Health condition

The health condition for which the COS is developed are fractures of the acetabulum.

#### Target population

The final COS will be applicable to studies of skeletally mature patients who have an acetabular fracture that is being surgically treated. For studies involving patients with pathological fractures, the COS will not be applicable as other outcomes could probably be of interest in these cases.

#### Intervention

Any type of surgical intervention for the treatment of acetabular fractures will be eligible, but nonsurgical interventions will be ineligible.

## Methods

This systematic review protocol was prepared in accordance with the COMET Handbook [9]. To the extent possible for a systematic review for the development of a COS, the protocol was reported according to the Preferred Reporting Items for Systematic review and Meta-Analysis Protocols (PRISMA-P) and the Core Outcome Set-STAndardised Protocol Items (the COS-STAP statement) [15, 16]. The PRISMA-P and COS-STAP Statement checklists are provided in Additional files 1 and 2. The COS for acetabulum fractures was registered with the COMET database (https://www.comet-initiative.org/Studies/Details/2123) and the protocol via PROSPERO (CRD42022357644).

### Eligibility criteria

#### Inclusion

Studies that examine skeletally mature patients (≥ 16 years) suffering from isolated acetabular fractures with or without fractures of the proximal femur who were treated surgically will be included. In order to identify all relevant outcomes, any prospective and retrospective clinical study that is examining at least 10 patients and that was published in English or German will be eligible.

#### Exclusion

Studies with purely nonsurgical treatment, multiple injured patients (e.g., *ISS* ≥ 16), juvenile patients (< 16 years/skeletally immature), or patients with pathological fractures will be excluded as in such cases different outcomes could be of interest. Case reports, biomechanical, cadaveric, and animal studies as well as studies with less than 10 patients will be excluded. Systematic reviews will be excluded to avoid duplications, but their bibliographies will be screened for possible eligible studies.

### Search strategy

The search will be performed on the following electronic databases and clinical trial registries from their inception to the present:

- MEDLINE via PubMed
- Cochrane Central Register of Controlled Trials (CENTRAL)
- Web of Science Core Collection
- ClinicalTrials.gov
- World Health Organization International Clinical Trials Registry Platform (ICTRP)

The terms “acetabulum” and “fracture” will be used to identify all outcomes reported in studies investigating surgical treatment of acetabular fractures. There will be no restriction regarding the publication date. Studies not published in English or German will be excluded. As an example, the search strategy for MEDLINE via PubMed is provided in Additional file 3.

### Study records

All search hits will be exported to the Covidence^®^ software [17]. After deduplication, the studies will be independently assessed for eligibility by two authors (D. S. & A. D.) [18]. First, the titles and abstracts of the studies will be screened followed by the full-text screening. The decision to include a study will be made according to the previously defined criteria. Any exclusion of a full text will be documented with reason. Disagreements will be solved by discussion. If no consensus can be achieved, a third reviewer (A. N.) will resolve the dispute. The selection process will be documented in a Preferred Reporting Items for Systematic Reviews and Meta-Analyses (PRISMA) flow diagram [19].

The data of the included studies will be extracted in a pre-designed form that will be piloted using five studies. If required, the data extraction form will be adjusted. Data extraction will be performed by two authors (D. S. & A. D.), independently [20]. Disagreements will be resolved through discussion, or if necessary, by a third reviewer (A. N.). The following data will be extracted: study characteristics (e.g., author(s), study design, recruiting country/countries, number of participants, characteristics of participants, and follow-up time) and outcome(s) (e.g., reported outcome(s), outcome definition(s), how the outcome(s) was measured, measurement instrument(s), and measurement timepoint(s)). Outcomes, their definitions, and the measurement instruments will be extracted literally. Where a validated patient-reported outcome measure (PROM) or performance outcome measures is used that is composed of multiple items, the following data will be extracted by the authors (if provided): verbatim name of the instrument and verbatim wording for each individual scale and item [21]. The frequency of use of each instrument will be noted. Data extraction will be carried out by using the Covidence^®^ software [17].

In case of missing data, the study authors will be contacted and asked for the missing data. If the study authors do not answer or do not provide the missing data, only the published data will be evaluated. The contact to authors and their answers will be documented.

### Risk of bias

As the objective of this systematic review is to identify all reported outcomes for surgically treated acetabular fractures rather than a quantitative synthesis of the study results, a comprehensive assessment of the risk of bias is not purposeful. However, the risk of outcome reporting bias will be assessed. For this purpose, the *Outcome Reporting Bias In Trials* (ORBIT) study classification system will be used. Thereby, for each outcome reported in the included studies, an outcome matrix will be created that distinguishes between full, incomplete, or no reporting. Based on this classification, the risk of bias due to the lack of inclusion of nonsignificant outcomes will be assessed. If it is certain that the reason for incomplete or missing reporting was other than statistically insignificant results, the risk of bias will be classified as not present. The risk of bias will be classified as low if it is presumed but not certain that the results were reported incomplete or not at all due to a reason independent of the results generated. Last, the risk of outcome reporting bias will be assigned as high if it is either certain or likely that results are reported incomplete or not at all because the difference in treatment effects was not statistically significant [22].

The risk-of-bias assessment will be performed in Microsoft Excel^®^ by two authors (D. S. & A. D.), independently [23]. Any discrepancies will be resolved through discussion. This assessment will not be used to exclude studies or outcomes. Instead, it aims to increase awareness of another problem in the area of outcome reporting that can be reduced with a COS [9].

### Data synthesis

Firstly, the identified outcomes will be grouped. Accordingly, outcomes with various definitions that measure the same outcome will be grouped under one outcome name. Subsequently, these outcome names will be categorized into outcome domains [9]. For this purpose, the outcome taxonomy developed by the COMET Initiative will be used, which contains 38 outcome domains with the core areas: (1) death, (2) physiological/clinical, (3) life impact, (4) resource use, and (5) adverse events [24]. Grouping of verbatim outcome definitions into outcome names and outcome names into outcome domains will independently be done by two authors (D. S. & A. D.) from multidisciplinary backgrounds with the use of Microsoft Excel^®^ [21, 23]. Any discrepancies will be resolved by discussion. The authors will summarize the results in a narrative synthesis through tables and graphs.

With the widespread implementation of computer tomography in the 1980s and the increase in surgical therapy with modern implants since the 1990s, the outcomes measured may have changed [25, 26]. Therefore, if practicable, the authors will conduct subgroup analyses and present the reported outcomes by publication year (before and after 2000), to facilitate the identification of trends in the reporting of outcomes. This may cause outcomes measured only prior to 2000 being considered outdated and thus inappropriate for a COS for surgically treated acetabular fractures.

## Discussion

The systematic review is currently ongoing. The literature search was started in November 2022. Title/abstract screening is completed, and full-text screening has begun. The estimated finalization of the systematic review is targeted for January 2025.

The final systematic review will not remain free of limitations. Since only outcomes already investigated in published studies on acetabular fractures are considered, there is the possibility of missing relevant outcomes. However, a comprehensive database search without specified restrictions regarding the publication date will be conducted to reduce the risk of missing outcomes that are regarded as important outcomes by the community in the field of acetabular fractures.

Regardless of the results generated, the final systematic review will be published in a peer-reviewed journal that is widely known in the field of traumatology. This shall ensure that a high number of experts will be informed about the heterogeneity of reported outcomes within studies investigating acetabular fractures and the development of a COS. Additionally, the study results will be published on the COMET database. The results will also be disseminated within the German Society of Traumatology, e.g., by presenting the results at the German Congress of Orthopaedics and Traumatology and at topic-related working groups.

### Supplementary Information

Additional file 1. PRISMA-P checklist.

Additional file 2. The COS-STAP statement.

Additional file 3. MEDLINE via PubMed search strategy.

## Data Availability

Not applicable.

## Abbreviations

CENTRAL: Cochrane Central Register of Controlled Trials
COMET: Core Outcome Measures in Effectiveness Trials
COS: Core outcome set
COS-STAD: Core Outcome Set-STAndards for Development
COS-STAP: Core Outcome Set-STAndardised Protocol Items
ICTRP: International Clinical Trials Registry Platform
ORBIT: Outcome Reporting Bias In Trials
PRISMA: Preferred Reporting Items for Systematic reviews and Meta-Analyses
PRISMA-P: Preferred Reporting Items for Systematic review and Meta-Analysis Protocols
PROM: Patient-reported outcome measure
