# Supplementary material for: Developing a core outcome set for acetabular fractures: a systematic review protocol
Source: Syst Rev. 2024 Jun 5;13:150. doi: 10.1186/s13643-024-02571-8 (PMC11151679; doi:10.1186/s13643-024-02571-8)
Supplement: Supplementary file 2 — Additional file 2. The COS-STAP statement. [file 13643_2024_2571_MOESM2_ESM.docx]

**The COS-STAP Statement**

# **This checklist has been changed from Table 1 in** Kirkham JJ, Gorst S, Altman DG, Blazeby JM, Clarke M, Tunis S, et al. Core Outcome Set-STAndardised Protocol Items: the COS-STAP Statement. Trials. 2019; 20:116. Epub 2019/02/11. doi: 10.1186/s13063-019-3230-x

| **Topic** | **Item number** | **Checklist Item** | | **Page number** |
| --- | --- | --- | --- | --- |
|  |  |  |  |  |
| TITLE/ABSTRACT | | | | |
| Title | 1a | | Identify in the title that the paper describes the protocol for the planned development of a COS | 1 |
| Abstract | 1b | | Provide a structured abstract | 2-3 |
| INTRODUCTION | | | | |
| Background and objectives | 2a | | Describe the background and explain the rationale for developing the COS, and identify the reasons why a COS is needed and the potential barriers to its implementation | 4-5 |
|  | 2b | | Describe the specific objectives with reference to developing a COS | 5 |
| Scope | 3a | | Describe the health condition(s) and population(s) that will be covered by the COS | 5-6 |
|  | 3b | | Describe the intervention(s) that will be covered by the COS | 6 |
|  | 3c | | Describe the context of use for which the COS is to be applied | 5 |
| METHODS | | | | |
| Stakeholders | 4 | | Describe the stakeholder groups to be involved in the COS development process, the nature of and rationale for their involvement and also how the individuals will be identified; this should cover involvement both as members of the research team and as participants in the study | Not applicable |
| Information sources | 5a | | Describe the information sources that will be used to identify the list of outcomes. Outline the methods or reference other protocols/papers | 6-10 |
|  | 5b | | Describe how outcomes may be dropped/combined, with reasons | Not applicable |
| Consensus process | 6 | | Describe the plans for how the consensus process will be undertaken | Not applicable |
| Consensus definition | 7a | | Describe the consensus definition | Not applicable |
|  | 7b | | Describe the procedure for determining how outcomes will be added/combined/dropped from consideration during the consensus process | Not applicable |
| ANALYSIS | | | | |
| Outcome scoring/feedback | 8 | | Describe how outcomes will be scored and summarised, describe how participants will receive feedback during the consensus process | Not applicable |
| Missing data | 9 | | Describe how missing data will be handled during the consensus process | Not applicable |
| ETHICS and DISSEMINATION | | | | |
| Ethics approval/informed consent | 10 | | Describe any plans for obtaining research ethics committee/institutional review board approval in relation to the consensus process and describe how informed consent will be obtained (if relevant) | Not applicable |
| Dissemination | 11 | | Describe any plans to communicate the results to study participants and COS users, inclusive of methods and timing of dissemination | Not applicable |
| ADMINISTRATIVE INFORMATION | | | | |
| Funders | 12 | | Describe sources of funding, role of funders | 12 |
| Conflicts of interest | 13 | | Describe any potential conflicts of interest within the study team and how they will be managed | 12 |
