# Supplementary material for: Developing a core outcome set for acetabular fractures: a systematic review protocol
Source: Syst Rev. 2024 Jun 5;13:150. doi: 10.1186/s13643-024-02571-8 (PMC11151679; doi:10.1186/s13643-024-02571-8)
Supplement: Supplementary file 3 — Additional file 3. MEDLINE via PubMed search strategy. [file 13643_2024_2571_MOESM3_ESM.docx]

MEDLINE via PubMed search strategy

1. (acetabul*[Title/Abstract]) OR (acetabulum[MeSH Terms])

2. (fracture*[Title/Abstract]) OR (fracture, bone[MeSH Terms])

3. #1 AND #2
